# Editorial: Concomitant pathogenic mutations in oncogene-driven subgroups: when next generation biology meets targeted therapy in NSCLC

**DOI:** 10.3389/fonc.2023.1239304

**Published:** 2023-06-22

**Authors:** Umberto Malapelle, Fabrizio Tabbò, Lucia Anna Muscarella

**Affiliations:** ^1^ Department of Public Health, University Federico II of Naples, Naples, Italy; ^2^ SC Oncologia ASLCN2 Alba e BRA, PO Michele e Pietro Ferrero, Verduno, Italy; ^3^ Laboratory of Oncology, Fondazione IRCCS Casa Sollievo della Sofferenza, San Giovanni Rotondo, Italy

**Keywords:** NSCLC, oncogene driver, co-occurrent mutations, targeted therapy, prognosis

Starting from the Epidermal Growth Factor Receptor (*EGFR*) (1) and Anaplastic Lymphoma Kinase (*ALK*) (2), and continuing with the identification of other well-known driver genes for target therapy in non-small-cell lung cancer (NSCLC), such as Proto-oncogene tyrosine-protein kinase ROS (*ROS1*) (3), v-Raf murine sarcoma viral oncogene homolog B (*BRAF*) (4), Kirsten rat sarcoma virus (*KRAS*) (5), Rearranged During Transfection (*RET*) (6), Tropomyosin receptor kinase 1-2-3 (*NTRK1-2-3*) (7), Neuregulin 1 (*NRG1*) (8), Erythroblastic oncogene B (*ERBB2*) (9), and MET proto-oncogene (*MET*) (10), all clinical trials carried out worldwide in the last decade aim to identify and evaluate the efficacy of different generations of target inhibitors (11, 12).

The impressive clinical efficacy of tyrosine kinase inhibitors (TKI) for oncogene-addicted subgroups of NSCLC has inevitably oriented the scientific community towards an oncogene-centric molecular classification paradigm of these tumors, where the identification of single or largely non-overlapping oncogenic driver events guides clinical decisions and forecasts patients’ responses (13). This approach allowed for oncogene-addicted NSCLC patients an unimaginable, but variable objective responses, progression-free survival and overall survival to up-front therapies (14). That was correlated at first instance to phenotypic variability and *de novo* resistance events, with rare observed complete responses (15–19).

Following the improvement in the knowledge of NSCLC genomic complexity, there was growing evidence that this monocentric model fails to adequately capture the clinical complexity of NSCLC and warrants revision to better modulate therapy in lung cancer patients. The use of molecular analysis techniques, most notably next generation sequencing (NGS), has revealed ever-increasing evidences that molecular intra-driver heterogeneity in tumors could guide the clinical heterogeneity. Beside of distinct effects of individual oncogene alleles (20–22) many under-investigated multiple non-random patterns of co-occurring mutations can be one of the main cause of the observed variations in response to therapies in NSCLC oncogene-dependent patients’ groups (23, 24), depending on several factors: the disease stage of tumors (25, 26), the selective pressure imposed by previous anticancer therapy, the clonal or subclonal nature as well as timing of co-alterations, immune-surveillance and –selection that could drive the oncogenic mutational landscape (27). The significance of such co-mutations as mediators of various NSCLC phenotypes has just lately come into attention, and existing molecular stratification frameworks do not adequately account for their functional influence (28).

The first and most supported evidences in this field were from the most frequent druggable genes of NSCLC, as *KRAS* and *EGFR*. The census of major *KRAS* co-mutations in advanced lung adenocarcinoma identified co-occurred lesions in a set of core gene including *LKB1, KEAP1, ATM* and *RBM10* that are related to early metastatic dissemination, tumor maintenance and an aggressive clinical phenotype in response to standard chemotehrapy, immunotherapy and biological agents (29). It is also crucial to remember that even genetic changes that do not exhibit statistically significant patterns of co-occurrence could nonetheless have crucial biological connections. For example, even though *TP53* mutations are less common in *KRAS* mutant lung adenocarcinoma than in other oncogene-driver subgroups, *TP53* inactivation is frequent and has a significant influence in this kind of cancer having an early-stage and chemo-refractory conditions or advanced PDL1-PD1 resistance profile (30, 31). A critical role of *TP53* mutations was also observed by analyzing its impact in NSCLC EGFRex20ins mutated patients, where *TP53* mutations was established as negative prognostic marker and also correlated to poor prognosis for EGFR ex20ins near-loop patients treated with second-/third-generation EGFR-TKIs, as well as copy number gain instability and higher tumor mutational burden (TMB) (32).

More recently, the co-mutations landscape and genomic architecture of lung adenocarcinoma driven by rare oncogenic alterations, such as *BRAF* mutations (33) or fusions involving *ALK, ROS1* and *RET* genes was highlighted. Compared with other driver subgroups, rearranged–positive NSCLC showed higher prevalence of *CDKN2A* and *CDKN2B* loss co-occurrent with *TP53* mutations (34) or MYC amplification (35, 36). By contrast, *TP53* mutations are underrepresented in NSCLC patients having MET exon 14 skipping, who frequently showed *MDM2* and *CDK4* co-occurrent amplifications events as an unfavorable outcome predictor (36).

Much more scientific advances are demanded to close the gap in this field and they must necessarily go through a re-evaluation of current clinical trials by including the genetic landscape of lung tumors.

Moreover, the identification of the clonal or subclonal architecture as well as the arising time of co-alterations may help to clarify the clinical complexity of NSCLC and reveal crucial details about their contributions to various stages of carcinogenesis (37), the nature of the microenvironment surrounding NSCLC (38), and its immune context (37). Due to all of these factors, it will be necessary in the future to compile a list of co-occurring pathogenic abnormalities in NSCLC, functionalize them, and assess their therapeutic value. This information will then be used to develop more specialized treatment plans that will translate in better clinical results for patients.

Actually, technology is helping us and the gold standard to identify co-occurring mutations is actually NGS on DNA/RNA extracted from tissue biopsy since it represents less time- and money-consuming approach to obtaining information about the molecular status of driver genes for target therapies (Yang et al.). In addition, the growing field of precision immunotherapy, the discovery of co-mutations in liquid biopsy, and the comparison of genetic results from analyses of tissues and blood will offer for personalized anticancer therapy challenges and opportunities (39). Multiregional sequencing analysis by large consortia has demonstrated the ability of ctDNA to capture the clonal structure from tumor tissue as well as to unveil additional heterogeneity at relapse when compared to tissue samples (37). The measurements of sub-clonal expansion in different clinical time may also enable to predict future metastatic sub-clones and give the chance to eradicate such clones months or even years before the clinical relapse of tumors.

## Author contributions

UM, FT and LAM: conceptualization, writing—original draft preparation, writing—review and editing, and supervision. All the authors have read and agreed to the published version of the manuscript.
